# Platelets’ Role in Dentistry: From Oral Pathology to Regenerative Potential

**DOI:** 10.3390/biomedicines10020218

**Published:** 2022-01-20

**Authors:** Serena Bianchi, Diana Torge, Fabiola Rinaldi, Maurizio Piattelli, Sara Bernardi, Giuseppe Varvara

**Affiliations:** 1 Department of Life, Health and Environmental Sciences, University of L’Aquila, 67100 L’Aquila, Italy; serena.bianchi@univaq.it (S.B.); diana.torge@graduate.univaq.it (D.T.); 2 Department of Innovative Technologies in Medicine & Dentistry, University of Chieti—Pescara ‘Gabriele d’Annunzio’, Via dei Vestini 11, 66100 Chieti, Italy; f.rinaldi0993@gmail.com (F.R.); maurizio.piattelli@unich.it (M.P.); gvarvara@unich.it (G.V.); 3 Center of Microscopy, University of L’Aquila, 67100 L’Aquila, Italy

**Keywords:** platelets, PRF, PRP, bleeding disorder

## Abstract

Platelets are a cellular subgroup of elements circulating in the bloodstream, responsible for the innate immunity and repairing processes. The diseases affecting this cellular population, depending on the degree, can vary from mild to severe conditions, which have to be taken into consideration in cases of minor dental procedures. Their secretion of growth factors made them useful in the regenerative intervention. The aim of this review is to examine the platelets from biological, examining the biogenesis of the platelets and the biological role in the inflammatory and reparative processes and clinical point of view, through the platelets’ pathology and their use as platelets concentrates in dental regenerative surgery.

## 1. Introduction

Platelets represent a cellular subgroup of the elements circulating in the bloodstream. A human adult has nearly one trillion circulating platelets, with a lifespan of 8–10 days [1]. These cellular circulating elements play important key roles: responding to vessel injuries, regulating angiogenesis, and the innate immunity [2]. 

**Figure 1:**
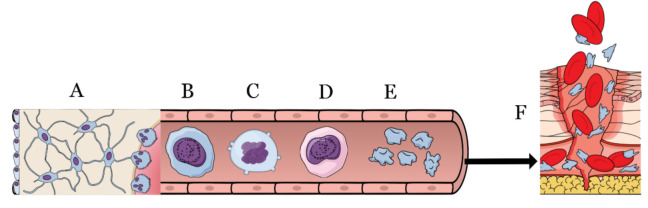
From the bone marrow (**A**) through the bloodstream, the megakaryoblast (**B**) evolves into megakaryocyte (**C**,**D**) to finally acquire the platelet form (**E**). Platelets are activated during the injures (**F**) by means of the coagulation cascade to block the blood loss and to start the very first steps of healing.

The functional roles in hemostasis, inflammation, and regeneration place the platelets as characters of human physiology (Figure 1); eventual pathologies affecting them influence the daily life of patients, as well as their survival in severe cases [3].

In dental medicine, platelets are studied and well-acknowledged due to the disorders that can affect the hemostasis [4] and the regenerative potential in oral surgery procedures [5].

From the place of their origin, the bone marrow, through the bloodstream, to the periphery, platelets’ journey is worthy of being examined. 

This review aims to go through the platelets’ biology, describing their origin, morphology, physiology, and pathologies relevant to oral surgery procedures and the related therapies. Finally, we examine the recent regenerative potentiality in dental surgery driven by the use of autologous platelet concentrates. 

### 1.1. Biological Characteristics of Platelets

Platelets are small, anucleate, and disc-shaped cells whose main structural elements are plasma membrane, open canalicular system (OCS), dense tubular system (DTS), a spectrin-based membrane skeleton, and actin-based cytoskeletal network. They also present a peripheral band of microtubules and several organelles, such as α-granules, dense granules, peroxisomes, lysosomes, and mitochondria [6].

The plasma membrane owns a bilayer of phospholipids, in which cholesterol, glycolipids, and glycoproteins are immersed. In this plasma membrane, sodium and calcium-adenosine triphosphate ensure the intracellular ionic environment of the platelet, while phospholipids’ task is to regulate the coagulation process. Other crucial components of the platelet plasma membrane are lipid rafts, cholesterol, and sphingolipid-rich microdomains, essential for signaling and intracellular trafficking. In addition, the platelet plasma membrane also includes surface receptors, which control signal-dependent platelet activation and participate in coagulation, inflammation, antimicrobial host defense, angiogenesis, and wound repair [7].

The OCS regulates the flux of external elements into the platelet, and at the same time, it is essential for the release of granule contents. Additionally, OCS is also an extensive internal membrane reservoir that promotes filopodia formation and platelet adhesion. Lastly, the OCS is a storage site for plasma membrane glycoproteins [7]. DTS is a closed-channel network of endoplasmic reticulum residues, whose primary function is to capture ionized calcium (Ca^2+^) supported by calreticulin, a calcium-binding protein. 

The cytoskeleton of inactive platelet is well-defined and highly specialized, which protects the morphology and integrity of platelets. Platelet cytoskeleton is composed of a spectrin-based membrane skeleton, an actin cytoskeleton, and a microtubule coil. Spectrin-based membrane skeleton consists of self-assembled or elongated spectrin strands that laminate the cytoplasmic surface of OCS and plasma membrane systems. In the actin cytoskeleton, forty percent of the actin subunits assemble to create 2000–5000 linear filaments in the resting platelets, while tubulin is equally distributed in dimer and polymer fraction in the resting microtubule coil [6,7].

Concerning granules distribution, in the inactive platelet, they are located near the OCS membranes, and during the activation process, they fuse and secrete into the OCS. In this context, platelets have two primary storage granules: α-granules and dense granules [7]. Alpha granules are the most widespread and most prominent secretory granules: according to their function, they present proteins essential for platelet adhesion during the vascular repair. With a 200–500 nm diameter, these granules are characterized by a spherical shape and a dark central core. They arise from the trans-Golgi network and include several membrane proteins essential for platelet function (e.g., P-selectin and CD36). Alpha granules also present over 30 angiogenetic proteins, regulators of the new blood vessel. Dense granules are 250 nm in size, visible on electron microscopy as dense bodies, with an electron-dense core. In this context, dense granules are helpful to collect additional platelets in the site of vascular damage, and they also contain active hemostatic molecules, such as catecholamines, serotonin, calcium, ADP, and ATP [1].

Platelets show a small number of mitochondria, valid for energy production. They also present lysosomes, which contain hydrolytic enzymes, including acid phosphatase, arylsulfatase, and peroxisomes, dispersed in their cytoplasm [6]. 

Daily platelets production starts from bone marrow megakaryocytes, rare myeloid cells, which represent less than 0.1% of the cellular population of the bone marrow. Megakaryocytes originate from pluripotent hematopoietic stem cells, which create burst and colony-forming precursors and are subjected to maturation. Thrombopoietin (TPO), Interleukin-3 (IL-3), stem cell factor (SCF), Interleukin-6 (IL-6), and Interleukin-11 (IL-11) are the main regulating factors of megakaryocytes’ differentiation process [8]. 

During the maturation process, megakaryocytes are subjected to endomitosis, a chromosomal duplication without cell division, which generates a unique polylobulated nucleus without completing the M phase. Ploidy is essential for megakaryocytes’ cytoplasmic volume, as it is determinant in lipid and protein synthesis, considered propaedeutic events for platelets’ production. Lipid and protein synthesis contributes to increasing megakaryocyte’s size (approximately to a diameter of 100 µm), supporting the production of platelet-specific organelles, granules, cytoskeletal proteins, and the invaginated membrane system, an extensive internal membrane reservoir, essential for platelet synthesis [6]. Regarding the maturation dynamics, during this process, megakaryocytes move from the osteoblastic to the vascular niche, a dynamic scaffold, which regulates megakaryocyte maturation and, consequently, platelets synthesis. 

Another significant stage, which contributes to the complex nature of platelet synthesis, is proplatelet elongation. This process starts with the erosion of megakaryocytes, which leads to the genesis of pseudopod-like structures that elongate tubular projections. A complex network of protein filaments regulates proplatelet elongation: the continuous polymerization of tubulin bundles determines the elongation process at their free plus end, and simultaneously, during this window of time, there is also the sliding of overlapping microtubules induced by dynein.

As regards the actin contributing to proplatelet production, limited evidence confirms its pivotal role in this process [1]. In addition, another significant regulator of platelets’ synthesis is the hematopoietic β–1 tubulin isoform, expressed by mature megakaryocytes that elicits an essential role not only in platelets’ production but also in the definition of their structure and function. 

Due to the migration process from the osteoblastic to the vascular niche, which involves several megakaryocytes, bone marrow stroma plays a fundamental role in megakaryocyte maturation is reserved for [6]. The bone marrow influences proplatelet synthesis by expressing specific matrix-receptor signals: type I collagen, for example, an extracellular protein widely diffused in the osteoblastic niche. Simultaneously, bone marrow prevents proplatelet synthesis and enhances megakaryocyte spreading. These dynamics are governed by the interaction between type collagen I and integrin α2β1, activating the small GTPase Rho and Rho-kinase ROCK. In addition, Rho-kinase ROCK is involved in the phosphorylation of the regulatory light chain of myosin IIA to reduce myosin ATPase activity and overpower proplatelet production. Moreover, among the most significant extracellular matrix proteins with a regulating potential on platelet production are type IV collagen, fibrinogen, and vitronectin [6].

In conclusion, proplatelet production results from a complex balance between molecular and protein factors, which influence the cellular environment of platelets and determine their varied biological function.

### 1.2. Platelets’ Activation

As previously described, platelets arise from megakaryocytes and are rapidly recruited during vessel damage to create a hemostatic clot in the vessel wall. Moreover, the platelet adhesion and aggregation process require specific platelet–endothelium and platelet–platelet interactions. The adhesion process is the first step of primary hemostasis: at this point, platelets interact with extracellular matrix proteins through adhesive glycoproteins (GP). Therefore, platelets’ activation results from a complex cascade of signals and receptor cross-linking, a process that induces significant changes in the assembly of cytoskeletal proteins [7]. At the same time, when the endothelium of a blood vessel is damaged, damaged cells produce endothelin, enhancing vessel contraction to stop blood loss. At this level, plasma glycoprotein von Willebrand factor (vWF) binds to the exposed collagen through GPIIb/IIIa receptors on the surface of the subendothelium as a substrate for platelet adhesion.

Alternative adhesion substrate in this step is also the modular glycoprotein thrombospondin-1 (TSP1), while among the most involved collagen receptors, there are GPIa/IIa, CD36, and GPVI, essential in promoting collagen-induced signaling [1,7]. 

After their activation, platelets release different granule components, which ensure the interaction between platelets and vascular cells [9]. Platelet dense bodies release secondary agonists, such as ADP and serotonin. ADP is considered a prominent amplifier of platelet activation, while serotonin exerts a procoagulant role. As regards serotonin, this vasoconstrictor binds to Gq-coupled 5HT2A receptor and enhances the platelet response through ADP. At the same time, the dense tubular system contains a Ca^2+^ pool, whose fluxes are essential for platelet activation, attraction, and aggregation. In this context, receptors expressed on platelets’ surface support platelets’ secretion and their irreversible aggregation: among them, the most significative are P2Y1 receptor, which regulates the mobilization of Ca^2+^ and morphology changes, and the P2Y12, essential in platelet secretion and irreversible aggregation [7]. The α-granules instead present large adhesive proteins (e.g., vWF, TSPI, vitronectin, fibronectin), but also mitogenic factors (e.g., PDGF, VEGF, TGFβ), coagulation factors (e.g., factors V, VII, XII, XIII), and protease inhibitors, released after the activation stage [1,7]. 

Another significant event for platelet physiology is their aggregation: this stage is characterized by the accumulation of platelets into a hemostatic clot and is completely regulated by GPIIb/IIIa, the central platelet receptor. The platelet stimulation during the aggregation induces an increase in GPIIb/IIIa, through α-granules exocytosis and activation of surface-exposed GPIIb/IIIa. The linkage between GPIIb/IIIa and its ligands promotes considerable molecular and morphological changes, leading to phosphorylation of tyrosines of the cytoplasmatic GPIIIa-chain [7]. Disulfide variations in the GPIIb/IIIa complex, induced by the disulfide isomerase, enhance high-affinity binding sites for fibrinogen, which usually binds discoid platelets during platelet–platelet contact. In the end, low-density lipoproteins (LDL) are also considered a ligand of GPIIb/IIIa, with potential regulative effects on platelet function. 

During secondary hemostasis, the generation of a platelet clot is determined by a thrombin-mediated conversion of fibrinogen to fibrin. At this stage, the platelet plasma membrane is essentially formed by phospholipids, cholesterol, and glycolipids. Among phospholipids, phosphatidylcholine (PC), sphingomyelin (SphM), phosphatidylethanolamine (PE), phosphatidylserine (PS), and phosphatidylinositol (PI) are located in the plasma membrane: specifically, SphM and PC are diffused in the outer left, while PE in the inner left [7]. The activation process of platelets is linked to a flip-flop move of PS, whose exposure on aggregated platelets supplies a catalytic surface for pro coagulation, preventing the synthesis of thrombin in the site of injury. The shedding of microvesicles from the platelet surface is due to the combination of collagen and thrombin: this process is determined by activation of calpain, induced by Ca^2+^, which promotes the formation of procoagulant vesicles.

At the same time, a stimulatory platelet signaling enhances the production of several intracellular messenger molecules: among them, Ca^2+^, products of the phospholipase C (PLC) phosphoinositol hydrolysis, diacylglycerol, inositol-1, 4, 5-triphosphate (IP3), and thromboxane (TxA2). ADP and TxA2 interconnect with seven transmembrane receptors associated with GTP-binding heterotrimeric G proteins to activate different signaling pathways. PLC activation is due to receptors coupled to the Gq family of G proteins, and this activation induces PIP2 hydrolysis, with mobilization of Ca^2+^ from the dense tubular system [1,7]. The increase in intracellular Ca^2+^ enhances the phosphorylation of the myosin light chain as an essential event for morphology change. Granule secretion is another significant response to Ca^2+^ mobilization, which determines the release of ADP from dense bodies: after this release, ADP links to P2Y12, supporting platelet activation. Regarding the TxA2 activation pathway, it is confirmed that TxA2 binds to Gq-coupled TP receptors to enhance the stimulatory process [1,7]. 

In the absence of a wound, prostaglandin I2 and nitric oxide (NO), released by the endothelial cells, represent platelet inhibitors. They promote an increase in cAMP through adenylate cyclase and guanylate cyclase, respectively, and promote a decrease in IP3 and Ca^2+^ levels, with a consequential decrease in platelet activation [7].

## 2. Platelets’ Pathologies Relevant for Oral Surgery

Platelets, therefore, play a crucial role in the coagulation cascade as constituents of factor X and prothrombin conversion complexes through the release of platelet factor 3 (PF3) [10]. In some cases, platelets may not complete the release of this factor due to defective thromboxane production because of the administration of anti-inflammatory drugs such as aspirin, NSAIDs, tricyclic antidepressants, lactam antibiotics, and calcium channel blocker drugs, but also due to a deficiency in the production of dense granule ADP [11]. While in healthy subjects, drug-induced impairment of platelet function has no clinical significance, in patients with coagulation disorders, uremic or thrombocytopenic patients, and those taking heparin or coumarin anticoagulants, drug-induced platelet dysfunction can cause severe bleeding [4]. Screening tests should therefore be carried out on these patients before any surgical procedure [4]. 

Structural or enzymatic alterations of platelet cause functional deficits and platelet disorders, which can be congenital or acquired [12].

Depending on the function that is compromised, we can distinguish [13]:

− Platelet diseases with an adhesion defect;
− Thrombocytopathies with an aggregation defect;
− Thrombocytopathies with a defect in the release reaction.

One of the causes of hemorrhagic diathesis, therefore, is platelet deficiency or dysfunction. The severity of hemorrhagic diathesis is very variable, concerning the varying degrees of platelopenia and the possible coexistence of a defect in platelet function. 

A reduction in the number of platelets, or thrombocytopenia, causes hemorrhages if the platelet count falls below 100,000 per mm^3^; however, spontaneous bleeding does not occur if the count does not reach a value of fewer than 20,000 platelets. Individuals with low platelet counts and no alterations in function do not bleed spontaneously but may experience prolonged bleeding during trauma, surgery, dental extractions, or biopsies [4].

Thrombocytopenias can be caused by [14]:

− Reduced platelet production: aplastic anemia, leukemia, vitamin B12, or folic acid deficiency cause a reduced development and faster destruction of bone marrow cells, including the megakaryocytes. In cases of aplastic anemia, which can be inherited or acquired, the bone marrow stem cells are incapacitated to proliferate and differentiate.

Dental management of patients affected by aplastic anemia includes an accurate medical history and an interdisciplinary approach with the cooperation of the hematologist. In cases of surgical intervention, the treatment should be performed on the transfusion day, and antifibrinolytics should be prescribed to control the bleeding. In cases of some forms of acute leukemia, dental treatment is recommended before starting the oncologic therapy.

− Destruction of platelets is due to [14]:
  - Immunological mechanisms: circulating antibodies or immune complexes can cause immune-mediated destruction of platelets following autoimmune, drug-induced, or HIV-associated diseases.
  - Mechanical damage: caused by platelets colliding with prosthetic heart valves, thrombi, or narrowed vessel walls.
  - Hypersplenism: due to increased platelet sequestration by the splenic phagocyte system.

### 2.1. Immunological Mechanisms: IMMUNE Thrombocytopenic Purpura (ITP)

The ITP is caused by the formation of anti-platelet autoantibodies (IgG) directed against the glycoproteins of the platelet membrane, which cause their destruction [15]. The diagnosis depends on excluding other causes of platelopenia, so it is a disease of exclusion. 

ITP can be chronic and may occur more frequently in adult women under 40 years of age, in whom immune-mediated platelet destruction may be due to systemic lupus erythematosus, AIDS, complications of drug therapies, or it may be idiopathic [15]. Bleeding from the skin may occur as spot hemorrhages (petechiae), which may coalesce into ecchymoses acute: this is a disease of childhood, with equal frequency in both sexes. Its onset is sudden and is preceded by a viral illness, with an interval of 2 weeks, but it usually resolves spontaneously.

Recently, some cases of ITP have been described after administration of mRNA SARS-CoV-2 vaccines and after adenovirus-based SARS-CoV-2 vaccine [16]. Most patients responded to treatment with corticosteroids and intravenous immunoglobulins (IVIG) but showed little benefit following platelet transfusion, as is the case with ITP. Furthermore, it is unclear whether these cases are self-limiting or will persist and lead to chronic ITP.

### 2.2. Immunological Mechanisms: Heparin-Induced Thrombocytopenia

This type of thrombocytopenia is caused by the immune-mediated destruction of platelets by heparin, which binds to platelet factor IV and changes its conformation [17]. At this point, the immune system no longer recognizes platelet factor IV, which, bound to heparin, is attacked by antibodies, resulting in the formation of immune complexes. These immune complexes, even under conditions of thrombocytopenia, can paradoxically cause thrombosis by activating the platelets [17].

### 2.3. Immunological Mechanisms: HIV-Associated Thrombocytopenia

Thrombocytopenia is the most common hematological manifestation in HIV infection due to reduced production and increased destruction of platelets [18]. Indeed, the CD4 molecule, the central HIV receptor, in megakaryocytes undergo apoptosis. In addition, HIV infection also induces hyperplasia and dysregulation of B cells, predisposing to the development of immune-mediated thrombocytopenia, characterized by the formation of anti-platelet antibodies [18].

### 2.4. Mechanical Damage: Thrombotic Microangiopathies

Thrombotic microangiopathies comprise a variety of clinical syndromes, including thrombotic thrombocytopenic purpura (TTP) and hemolytic uremic syndrome (HUS) [19]. Both are characterized by fever, thrombocytopenia, microangiopathic hemolytic anemia, and renal failure, more severe in HUS [14]. Transient neurological deficits are also present in TTP, absent in HUS, which has a higher incidence in children. A common feature of both conditions is the widespread formation of hyaline thrombi within the microcirculation, which causes platelet consumption (thrombocytopenia) and microangiopathic hemolytic anemia due to the clash of red blood cells with these thrombi [19]. However, the pathogenetic mechanism responsible for thrombus formation is different. In TTP, there is a deficiency of an enzyme known as ADAMTS 13 or “von Willebrand factor metalloproteinase”, which degrades von Willebrand factor multimers, representing the form in which it circulates in the blood. In the absence of this enzyme, von Willebrand factor multimers accumulate in the plasma and promote the formation of platelet microaggregates in the microcirculation, causing TTP. In HUS, the cause is gastroenteritis caused by *E. coli*, which produces a toxin that binds to endothelial cells in the renal glomerulus and other organs, damaging them and initiating platelets activation and aggregation [19]. HUS can also affect adults, following pathologies that cause endothelial damage (drugs, radiotherapy). TTP and HUS are differentially diagnosed with disseminated intravascular coagulation (DIC) since they are all characterized by microvascular occlusion and microangiopathic hemolytic anemia [19], but in the former, activation of the coagulation cascade does not play a primary role; therefore, coagulation tests such as PT and PTT usually show typical values.

## 3. Hemorrhagic Diseases of Impaired Platelet Function

Qualitative defects in platelet function may be congenital or acquired. Acquired disorders mainly include ingestion of aspirin and other non-steroidal anti-inflammatory drugs [20] due to irreversible inhibition of cyclooxygenase, an enzyme required for thromboxane A2 synthesis, and uremia, during which numerous alterations in platelet function have been observed. However, the pathogenesis of bleeding during uremia is complex and not well understood.

Congenital disorders of platelet function, on the other hand, can be due to:

− Defects in platelet adhesion to the subendothelial matrix [21], as in the case of von Willebrand disease or Bernard-Soulier syndrome [22], an autosomal recessive disorder caused by the hereditary deficiency of the membrane glycoprotein complex Ib-IX, which acts as a receptor for von Willebrand factor, essential for the physiological adhesion of platelets to the subendothelial matrix.

Von Willebrand disease is classified as the most common inherited bleeding disorder due to an inherited defect that causes both deficits in platelet function and the coagulation cascade [23]. Indeed, the von Willebrand factor is crucial in platelet adhesion to the exposed extracellular matrix following vascular injury and in transporting coagulation factor VIII, a component of the intrinsic pathway required for factor X activation and ensuring its stability [24]. The half-life of circulating factor VIII is approximately 12 h if von Willebrand factor levels are standard but only 2–4 h if von Willebrand factor is defective or deficient, with alteration of the coagulation cascade [24]. Unbound factor VIII is therefore destroyed in the circulation.

Depending on the severity of the genetic expression, the disease has several variants, most of which are transmitted as autosomal dominant traits (types 1 and 2) [25]. The different variants of von Willebrand disease tend to cause mild to moderate clinical bleeding problems: notably, in mild cases, bleeding occurs only after surgery or trauma; in severe cases, spontaneous nosebleeds or bleeding of the oral mucosa may be noted [25]. 

Among the variants of von Willebrand disease [26], we can distinguish:

- Diseases associated with a reduction in circulating von Willebrand factor:
  - Type 1 (70–80% of cases) is the most common form of von Willebrand disease, due to non-sense mutations, with mild but variable clinical manifestations: the more significant the vWF deficiency in type 1 disease, the more likely we are to find signs and symptoms of hemophilia A [26].
  - Type 3 is a rare autosomal recessive form characterized by severe clinical manifestations caused by frameshift mutations or deletions [26].
- Diseases associated with qualitative deficiencies of von Willebrand factor:
  - Type 2, responsible for 15–20% of cases, is an autosomal dominant form due to non-sense mutations causing defective assembly of the multimers, with mild to moderate symptoms. Treatment involves desmopressin, which stimulates vWF, or factor VIII and vWF concentrate [26].
  - Defects in platelet aggregation, such as Glanzmann’s thrombasthenia, an autosomal recessive disorder characterized by a deficiency or malfunction of glycoprotein IIB-IIIa, a protein complex that contributes to the formation of “bridges” between platelets [26].

Platelet secretion disorders are characterized by a normal initial response to the aggregating stimulus of collagen but by a defect in the secretion reactions of thromboxane and the release of ADP in the granules [14].

Hereditary thrombocytopathies, therefore, derive from genetic alterations that lead to a deficiency in the number of platelets: there are diseases transmitted by a “dominant” and “recessive” mechanism [27]. 

In the first case, it is enough for only one of the two genes inherited from the parents to be defective for the development of thrombocytopenia. The recognition of the genetic nature of the defect is generally easy thanks to the medical history, which reveals the father or mother is also ill and, often, that other blood relatives have the same defect [27]. 

In the second case, the mutation is less serious and induces thrombocytopenia only when both genes are defective: the parents are “healthy carriers” of the disease, and only the son shows thrombocytopenia [27]. 

Finally, “sex-linked recessive” transmission is rare and occurs when a mother is a carrier, and only the male child/children develop the disease [27]. 

The clinical conditions derived from platelets’ defects are represented by various bleeding manifestations, from mild to moderate to very severe, affecting the skin, oral and nasal mucosa, intestine, and uterine mucosa [14]. In case of mucosal bleeding manifestations, with average platelet count, it is advisable to refer to the specialist center to perform bleeding time and in vitro platelet function tests (Born platelet aggregation or PfA-100 test) and perform the differential diagnosis with von Willebrand disease [14].

In order to reduce both the risk of gum disease and the occurrence of dental diseases requiring gum surgery, it is necessary to pay great attention to oral hygiene from an early age and prevent, in general, traumas and injuries [4]. 

## 4. Role of Platelets Derivates in Regenerative Dental Procedures

In dentistry, platelets find various applications in oral and maxillofacial surgery due to the possible use as a grafting material for bone rehabilitation to improve bone regeneration, especially in bone volume augmentation [28]. Regenerative dentistry represents a discipline continuously in evolution, where not only platelets and dentinal derivates are used as autologous biomaterial, but also the experimental use of stem cells in vitro studies gave promising results [29,30]. 

As known, the repair or regeneration of damaged tissue is a biological process in which various factors are involved, such as immune cells, growth factors, proteins, and in which we can distinguish four main phases [31]: hemostasis, inflammation, proliferation, and maturation.

The whole process is very complex, as it requires the combination of many factors and the perfect timing of their activation [31].

The technology advancement and the available centrifuges allowed the possibility to obtain platelet concentrates on an outpatient basis: the centrifugation of the blood taken from the patient separates the plasma part, rich in platelets, for autologous therapeutic use [32]. The fractioned blood is used to obtain both platelet gel (with a higher concentration of platelets than physiological) and fibrin glue with a high adhesive and hemostatic property [32].

The two most common platelet concentrates are platelet-rich plasma (PRP) and platelet-rich fibrin (PRF), which represent the first and second generation (Table 1), respectively [33]. 

The PRP is obtained from the heparinization, which makes it incoagulable of the blood taken from the patient and can be stored for a few days at −19 °C, or it can be prepared immediately after the blood collection [35]. In any case, before centrifuging the blood (the protocol includes two centrifugations) to remove the red blood cells, anticoagulant factors such as bovine thrombin or CaCl_2_ must be added to trigger the coagulation and fibrin formation cascade [35]. This process leads to a tumultuous fibrin network formation process, resulting in somewhat irregular fibrin [35]. PRP should also be used within 4 h of isolation, as growth factors are secreted within 10 min of preparation and reach 95% after 1 h [35]. 

This type of platelet concentrate presents disadvantages [36]:

- The presence of anticoagulants such as bovine thrombin could cause allergic reactions and coagulopathies due to the action of antibodies against factors V, XI, and consequent thrombus formation [36];
- Final preparation without rigidity requires the further addition of bone grafts to maintain a stable volume [36].

The PRF belongs to a new generation of hemo-concentrates obtained by the sole centrifugation, which do not require additives such as heparin or thrombin, allowing a slow release of growth factors [37]. Therefore, the blood is not manipulated in any way, and the obtained PRF must be used at the same time as its production since it is not storable [37]. The fibrin formation process is much more regular, and the cytokines and the growth factors remain trapped in its structure, making it stronger and more elastic and considerably increasing regenerative capacity [37,38].

PRF has the following bioactive properties:

- Stimulation, through the growth factors contained within it, of the proliferation, differentiation, chemotaxis, and adhesion of stem cells, promoting angiogenesis and immune processes [34];
- Increased expression of alkaline phosphatase (ALP) in stem cells, leading to faster mineralization of the newly formed tissue [34];
- Induction of mineralization of the defect, thanks to the growth factors it contains (TGF-β1 and PDGF) [39];
- The creation of an epithelial barrier by the PRF membrane [40].

The capacity for clot formation and organization is increased, thanks to the formation of a regular fibrin network, which provides an osteoconductive scaffold suitable for neo-angiogenesis and cell migration, which is essential for the bone apposition process [34,35,38,39,40]. 

This increases the speed of healing and the quality of the newly formed tissues.

The different types of PRF preparation found in the literature are based on different relative centrifugation forces (RCF).

Choukroun’s L-PRF or leukocyte-PRF (solid PRF) protocol includes [37]:

- A blood sample (10 to 100 cc of blood) taken and placed in a 10 mL glass tube (not plastic as the latter activates fewer coagulation factors);
- Centrifugation at 3000 rpm for 10 min.

This centrifugation produces a total concentration with a high percentage of growth factors, immune cells, and platelets that release important cytokines. 

At the end of centrifugation, the patient’s blood is separated into three phases:

- Superficial layer: cellular plasma;
- Middle layer: fibrin + platelets + leucocytes (PRF);
- Deep layer: blood cells.

The intermediate layer takes the form of a gelatinous substance, which can be used either as a membrane or as an adjunctive biomaterial [37]. The L-PRF does not have rigidity and always requires the support of a filler (even crushed PRF). In addition, it does not need to be cut or sutured: its consistency allows it to adapt perfectly to the bone. In clinical practices, the L-PRF is placed between two surfaces (even between two glass plates used to mix the cement, wrapped in sterile gauze) and exerts pressure with their weight for a certain period, transforming it into a resorbable membrane. Due to its rapid resorption time, the L-PRF represents a “reservoir” of growth factors [39].

The Ghaanatis PRF (also known as advanced PRF or A-PRF) is a solid PRF more malleable than the L-PRF. It is obtained using a reduced centrifugal force, 208 G on plastic tubes (whereas in the previous PRF, it is 708 G on glass tubes) [41]. 

The main advantages include a high concentration of leukocytes (due to slow centrifugation), a high concentration of platelets and neutrophil granulocytes, and a good release of different growth factors [41]. The matrix is more porous, allowing a better angiogenesis and blood vessel penetration; the soft consistency allows different use of the material such as:

- Pellets for bone grafting;
- Membrane.

The I-PRF is a platelet concentrate in the liquid form obtained using centrifugal force 60 G (at higher speed for less time), so no coagulation takes place [42]. 

Due to the lower centrifugation speed and time, it contains a high concentration of leukocytes and blood plasma proteins, and it can be used in different ways, such as injection into the deep tissue space or mixed with other graft materials [42].

The main advantages represented by the platelet concentrations are the contained growth factors [43]. The growth factors are released by platelets, macrophages, and leukocytes, which play a role in inflammation and wound healing by stimulating or inhibiting the mechanisms of migration, adhesion, proliferation, and differentiation [43]. They can exist in two forms: active and inactive and have short biological half-lives. 

Growth factors are linked to the different types of cells that secrete or stimulate the release of these factors [44]. Depending on the centrifugation, therefore, a different percentage of growth factors can be obtained in different types of platelet concentrate [44]. 

Among the various growth factors, mention must be made of the following [43]:

- Endothelial growth factor (EGF): it stimulates endothelial cell chemotaxis, mesenchymal cell mitosis, epithelialization and increases tissue tensile strength [43].
- Transforming growth factor β1 (TGF-β1): it regulates angiogenesis, upregulates vascular endothelium growth factor (VEGF), regulates the tissue repair process, immune modulation, synthesis of extracellular matrix, and plays a key role during bone regeneration, stimulating the chemotaxis and mitogenesis in osteoblast precursors and the deposition of mineralized tissue on the bone collagen matrix [43].
- Platelet-derived growth factor (PDGF): this factor is mainly produced by platelets, and it can be in the fibrin matrix in high quantities. PDGF regulates the migration, the proliferation of mesenchymal cells, stimulates collagen production, the mitogenesis of osteoblasts, fibroblasts, smooth muscle cells, and glial cells [43].
- Insulin-like growth factors (IGF): the IGF is secreted by platelets and over-regulates the proliferation and differentiation of various cells involved in tissue repair mechanisms, the differentiation and proliferation of mesenchymal cells, and at the same time, it is a regulator of programmed apoptosis [43].
- VEGF: macrophages and thrombocytes secrete VEGF, the main factor in stimulating angiogenesis, modulating tissue remodeling, and when added to bone materials, stimulates the formation of new bone tissue [43].

### Clinical Use of Platelets Derivates

Several studies and literature reviews, due to the regenerative properties mentioned above, have highlighted the effectiveness of the regenerative potential of these preparations [45].

The clinical applications of these preparations are in various areas such as guided periodontal and bone regeneration, sinus floor elevation, and support for the regeneration of the pulp-dentine complex [46,47,48]. However, there is no shortage of studies highlighting the limited effects of, for example, the efficacy of PRP in bone formation during sinus floor elevation procedures, as noted in the systematic review by Pocaterra et al. [49].

PDGF has also been used, in combination with advanced surgical techniques, to treat infrabony defects, as shown in the study by Del Fabbro M. et al. [50]. The study evaluated the effects of autologous platelet concentrates together to open periodontal surgery, with or without bone grafting, guided tissue regeneration (GTR), or in addition or without enamel matrix derivatives. However, the authors did not find that the addition of autologous platelet concentrates (APCs) to specific surgical techniques such as open flap with or without bone graft, or GTR or interventions with the presence of enamel matrix derivatives, would improve pocket depth, clinical attachment level, or radiographic results of filling the infraosseous defect, due to the high risk of bias in performing their revision [50].

APCs have also been used in the treatment of furcation defects, as shown in the review by Panda et al. [51], achieving an improvement in horizontal and vertical clinical attachment loss and furcation depth, in addition to open surgery and bone grafting, but not with GTR and enamel matrix derivatives. Furthermore, again regarding the treatment of furcation defects, the study by Tarallo et al. [46] demonstrated the effectiveness of the additional use of PRF to open periodontal surgery to improve periodontal regeneration, particularly in the presence of grade II furcation. 

In addition to regenerative properties, it has been shown in other reviews by Del Fabbro et al. [52,53] that platelet concentrates provide additional benefits during post-surgical healing, such as reduced pain and improved quality of life for patients.

Recently, the clinical efficacy of PRGF in addition to non-surgical periodontal therapy, such as scaling and root planing (SRP) in patients with periodontitis was evaluated by Panda et al. [51]; the authors concluded that the use of PRGF proved effective in both reducing pocket depth and bleeding index and gaining clinical attachment level. 

L-PRF has also been used in the treatment of single and multiple gingival recessions in conjunction with the coronal repositioning flap (CAF) technique; the efficacy of this, compared with CAF alone and with the addition of connective graft, was the subject of the study by Mancini et al. [47], who concluded their review by reporting that there were statistically significant results in mean root coverage, reduction in recession, gain in keratinized tissue width, gain in gingival thickness, patient perception of pain and discomfort with the use of L-PRF compared to CAF alone, but not in relation to the addition of connective graft in single and multiple CAF. The latter, however, showed statistically significant results only in relation to the patient’s perception of pain and discomfort. In recent years, PRP has also gained interest in regenerative endodontics, particularly in pulpotomy and apical surgery, being considered an ideal scaffold in this field. Indeed, autologous PRP acts as an ideal natural three-dimensional scaffold, supporting cell growth and differentiation and releasing various growth factors that influence tissue regeneration. The study by Sequeira et al. [48] evaluated the potential of using human apical papilla stem cells (SCAP) embedded in a PRP scaffold for endodontic regenerative procedures, in combination with ProRoot MTA or Biodentine. The results of this study provided evidence of de novo pulp and dentin-like tissue formation when SCAP and PRP were applied in the empty root canal space of root portions filled with cultured cells after they were isolated from apical papillae and implanted in the subcutaneous space of immunodeficient rats [48]. The results derived from the study are probably due to the biophysical properties of the existing dentin interacting with SCAP-derived odontoblasts or the increased concentration of bioactive molecules on the root dentin wall [48]. In addition to the presence of pulp tissue, blood vessels, and nerve fibers, a typical layer of odontoblast-like cells could also be observed in this study’s pulpal part of the new dentin tissue [48].

Finally, although no specific clinical studies have been conducted to date, there is substantial preclinical evidence of the antimicrobial effect of autologous platelet concentrates against certain species commonly found in the oral cavity, suggesting that they may be a valuable tool for controlling the onset of post-surgical infection [54,55].

## 5. Conclusions

From the bone marrow to the wound through the vascular supply, platelets have a crucial role in hemostasis, immune modulation, and repair mechanisms. 

The described diseases affecting platelets production and quality, with also severe consequences on the survival and patients’ quality of life, prove the unicity and the indispensability of these small elements and the necessity of an accurate medical history also in cases of dental surgery procedures. 

In addition, the secretive property of growth factors and of fibrin network, whose physical characteristics can vary and be artificially controlled, made them an important source in regenerative medicine. 

The fibrin network and the growth factors give to the platelets concentrates inductive and conductive properties and, therefore, a potential aid in regenerative bone procedures. 

## Figures and Tables

**Table 1:** Resuming table of the properties of PRP and L-PRF.

Blood Product	L-PRF	PRP
Protocol	2700 rpm for 12′	2400 rpm 10′
Flow	One step continuous	Two steps cloth activation
PDGF levels (ng/mL) [32]	High	Low
VEGF levels(ng/mL) [34]	Highest	Low
TGF-b 1 (ng/mL) [34]	High	Low
Reproducibility	No bias	Possible bias
Use of anticoagulants	No	Yes
Fibrin density	High	Low
Speed of fibrin formation	High	Low
Fibrin morphology	Tetramolecular	Tetramolecular
Handling	Easy	Complex

PDGF—platelet-derived growth factor, VEGF—vascular endothelial growth factor, TGF-β 1—transforming growth factor β 1.

## Data Availability

Not applicable.
